# Management of Chronic Obstructive Pulmonary Disease (COPD) Exacerbations in Hospitalized Patients From Admission to Discharge: A Comprehensive Review of Therapeutic Interventions

**DOI:** 10.7759/cureus.43694

**Published:** 2023-08-18

**Authors:** Khizar S Khan, Sanyah Jawaid, Unaib Ahmed Memon, Tharindu Perera, Usman Khan, Umm E Farwa, Urmi Jindal, Muhammad Sohaib Afzal, Waleed Razzaq, Zain U Abdin, Uzzam Ahmed Khawaja

**Affiliations:** 1 Basic Sciences, Foundation University Medical College, Islamabad, PAK; 2 Internal Medicine, Liaquat National Hospital and Medical College, Karachi, PAK; 3 Internal Medicine, Liaquat University of Medical and Health Sciences, Hyderabad, PAK; 4 General Medicine, Grodno State Medical University, Grodno, BLR; 5 General Practice, Akhtar Saeed Medical and Dental College, Lahore, PAK; 6 Emergency Medicine, Jinnah Sindh Medical University, Karachi, PAK; 7 Internal Medicine, KJ Somaiya Medical College, Mumbai, IND; 8 Medicine, Louisiana State University Health Sciences Center, Shreveport, USA; 9 Internal Medicine, Services Hospital Lahore, Lahore, PAK; 10 Medicine, District Head Quarter Hospital, Faisalabad, PAK; 11 Pulmonary and Critical Care Medicine, Jinnah Medical and Dental College, Karachi, PAK; 12 Clinical and Translational Research, Dr Ferrer BioPharma, South Miami, USA

**Keywords:** chronic obstructive pulmonary disease exacerbation, chronic obstructive pulmonary disease(copd), copd: chronic obstructive pulmonary disease, clinical outcomes, non invasive ventilation, invasive ventilation, therapeutic interventions, copd exacerbation

## Abstract

Chronic obstructive pulmonary disease (COPD) is a common and debilitating condition that often necessitates hospitalization for exacerbations. Since COPD exacerbations can cause significant morbidity and mortality, managing them is crucial for patient care. Effective management of COPD exacerbations is essential to prevent complications, as COPD exacerbations are associated with increased healthcare costs and decreased quality of life. This review aims to comprehensively discuss the management of COPD exacerbations, covering various pharmacologic and non-pharmacologic strategies. These include inhaled bronchodilators, systemic steroids, antibiotics, invasive and non-invasive ventilation, oxygen therapy, smoking cessation, immunization with pneumococcal vaccine, inhalers at discharge, pulmonary rehabilitation, long-term oxygen therapy (LTOT), ambulatory oxygen therapy, short-burst oxygen therapy, extracorporeal membrane oxygenation (ECMO), lung volume reduction surgery (LVRS), endobronchial procedures, and lung transplant. It is drawn upon various sources, including clinical studies, systemic reviews, and observational studies, to provide a comprehensive overview of current practices and identify areas for future research and innovation in managing COPD exacerbations. Addressing these areas of interest can improve patient outcomes and quality of life.

## Introduction and background

Clinical presentation, diagnostic criteria, and factors leading to chronic obstructive pulmonary disease (COPD) exacerbation

Chronic obstructive pulmonary disease (COPD) is often preventable and treatable, characterized by persistent airflow limitation, chronic inflammation, and progressive lung disease. Chronic obstructive pulmonary disease is comprised of emphysema and obstructive bronchiolitis. A patient can present with either or both of these manifestations simultaneously. Previously, chronic bronchitis was considered a type of COPD but has recently been replaced by obstructive bronchiolitis [1]. Chronic obstructive pulmonary disease is a persistent and advancing disease in which an individual may experience sporadic acute exacerbations characterized by respiratory symptoms that worsen beyond the typical presentation during a regular day. These exacerbations have a cumulative effect on the overall function of the respiratory system [2]. These exacerbations of COPD can result in hospitalization, a decrease in lung function, and increased mortality. According to the World Health Organization (WHO), COPD is the third leading cause of death worldwide, causing 3.23 million deaths in 2019 [3]. Unfortunately, it is expected to be the leading cause of death worldwide by 2030 [4]. Therefore, the effective management of COPD exacerbations is crucial to improving the quality of life and reducing the burden of the disease. Numerous guidelines and recommendations exist for managing COPD exacerbations; however, the optimal approach is still under debate. Managing COPD exacerbations is a complex and multifaceted process requiring a personalized approach based on the patient's clinical characteristics and comorbidities.

Chronic obstructive pulmonary disease is primarily seen in individuals aged 40 and above, usually in individuals who smoke [5]. These patients often present late, when significant damage to the airway tract has already occurred. Patients with COPD can present with several physical findings listed below:

A) Shortness of breath (dyspnea): Patients may experience breathlessness, especially during physical activity or exertion. These patients naturally adopt a sitting, forward-leaning position with their hands on their knees during episodes of respiratory distress, known as the tripod position. This position helps alleviate dyspnea by fixing and lifting the shoulder girdle, improving the length-tension relationship of accessory muscles, restoring the diaphragm's normal shape and function, decreasing the recruitment of specific muscles, and improving thoracoabdominal movement [6]. However, according to a study conducted in 2009, no significant difference was observed in COPD patients in the tripod position compared to sitting or supine positions [7].

B) Wheezing: Wheezing is a high-pitched whistling sound that occurs during breathing due to the narrowing of the airways. Unforced wheezing has a likelihood ratio of 2.6 for COPD diagnosis [6].

C) Cough: A persistent, productive cough consisting of sputum or phlegm is a common symptom of COPD.

D) Increased sputum production: Patients with COPD may produce more mucus than normal, which can cause difficulty breathing. Furthermore, this excess sputum plays a role in developing both temporary respiratory infections and the pathogenesis of various respiratory diseases, worsening COPD [8].

E) Cyanosis: Blue or grayish skin discoloration, especially around the lips and fingernails, due to low oxygen levels in the blood.

F) Barrel-shaped chest: Patients with COPD may have a rounded and bulging chest due to air trapping in the lungs. In most cases, the curvature of the spine, known as dorsal kyphosis, and the horizontal orientation of the ribs are observed. Other physical features include a prominent sternum, raised clavicles, a shortened neck, and wider spaces between the ribs. Developing a barrel-shaped chest is common in severe emphysema while aging alone can result in a similar chest deformity without lung disease [6].

G) Use of accessory muscles: Patients may use their neck, shoulder, and chest muscles to assist with breathing, especially during exertion. Despite the limitation in airflow, most patients employ both their expiratory and sternomastoid muscles. The expiratory muscles cannot decrease lung volume due to the airflow constraint [9].

H) Reduced breath sounds: Breath sounds at the mouth may be diminished or absent due to air trapping in the lungs. Patients with emphysema typically have quiet breathing at the mouth because the disease does not cause bronchial narrowing [6].

I) Decreased lung function: Pulmonary function tests (PFTs) may reveal decreased lung function, including decreased forced expiratory volume in one second (FEV1) and forced vital capacity (FVC). However, according to a study, if patients get regular treatment during the cycles of remissions and exacerbations, the decrease in FEV1 and FVC is significantly less [10].

**Figure 1:**
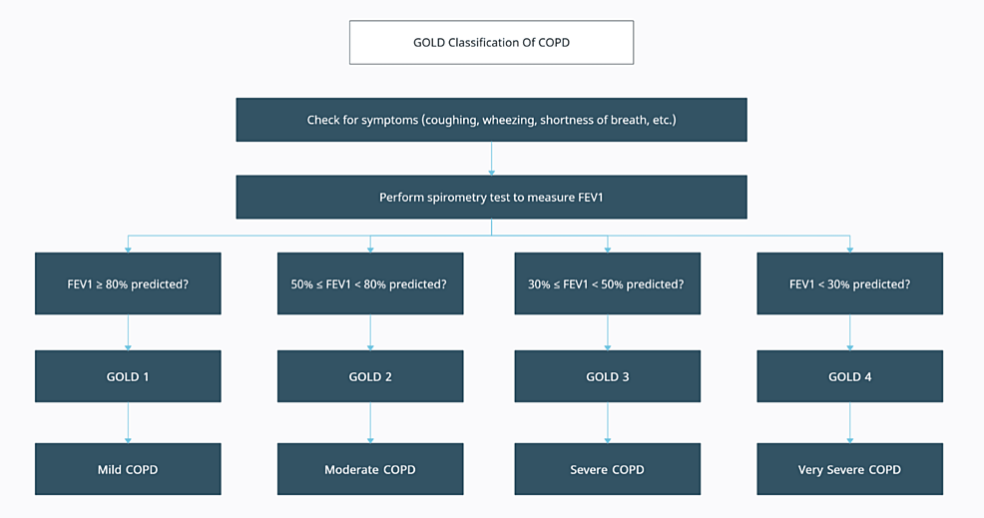
The GOLD classification of COPD Reproduced under the terms of the Creative Commons Attribution license. GOLD: Global Initiative for Chronic Obstructive Lung Disease; FEV1: forced expiratory volume in 1 second; COPD: chronic obstructive pulmonary disease [11]

It is important to note that not all patients with COPD will exhibit all of these physical findings, and some patients may exhibit additional symptoms and signs depending on the severity of their disease. The diagnosis of COPD is usually made by a combination of patient history, physical examination, PFTs, and imaging studies. According to the Global Initiative for Chronic Obstructive Lung Disease (GOLD) Report of 2023, confirmation of COPD diagnosis is achieved through the identification of non-fully reversible airflow limitation using spirometry, where the FEV1/FVC ratio after bronchodilation is <0.7 [11]. The Global Initiative for Chronic Obstructive Lung Disease classifies obstruction in COPD patients into four stages, as shown below and in Figure 1:

GOLD 1: Mild; FEV1 ≥80% predicted

GOLD 2: Moderate; 50% ≤ FEV1 <80% predicted

GOLD 3: Severe; 30% ≤ FEV1 <50% predicted

GOLD 4: Very severe; FEV1 <30% predicted [11, 12].

However, COPD can often be misdiagnosed, and the disease can advance before it is clinically detected. For this purpose, spirometry can detect COPD before symptoms become apparent [13]. Other lung function tests can also be performed, such as peak expiratory flow (PEF) tests, fractional exhaled nitric oxide (FeNO) tests, and arterial blood gas (ABG) analyses. Lung imaging modalities can also help in the diagnosis of COPD. These include chest X-rays and computed tomography (CT) scans [13]. According to epidemiological studies, a significant percentage of individuals globally suffering from COPD have never smoked, ranging from 20% to 40%. Therefore, while smoking remains the primary risk factor for COPD, other factors should also be considered [14]. There are many factors contributing to COPD. These include:

A) Smoking: This is the most significant risk factor for COPD. About 85% to 90% of all COPD cases are caused by smoking [15]. According to a study conducted in India in 2021, smokers exhibited a greater degree of dyspnea, more advanced COPD, reduced FEV1 after using a bronchodilator, and a more significant number of emphysematous alterations on X-ray imaging [16].

B) Exposure to air pollution: In COPD patients, exposure to outdoor air pollutants is linked to a decline in lung function and a rise in respiratory symptoms. Moreover, outdoor air pollutants are related to more frequent COPD flare-ups and higher mortality rates [17].

C) Temperature: There is a connection between extreme temperatures, whether hot or cold and elevated respiratory morbidity in COPD [17].

D) Passive smoking: Inflammation caused by second-hand smoke through cells and mediators, combined with the production of reactive oxygen species, destroys the alveolar walls. This suggests that second-hand smoke could be a potential cause of emphysema through this mechanism [18].

E) Genetic alpha-1 antitrypsin deficiency: This is commonly underdiagnosed and causes liver and lung damage. If the patient shows signs of liver damage and typical symptoms of COPD, one should order a blood test to rule out the possibility of alpha-1 antitrypsin deficiency [19].

F) Impaired lung growth during childhood: Various early-life exposures can decrease lung development during childhood, thus playing a role in developing COPD in later life [20]. Tobacco exposure to the child as far back as life in utero can also play a part in the development of COPD [21].

G) Gender: Females tend to exhibit symptoms of emphysema at earlier stages and with less exposure to tobacco compared to males, which can contribute to the development of the disease [22].

H) Non-adherence to treatment plan: Failure to follow prescribed medications, not using oxygen therapy as prescribed, or not attending pulmonary rehabilitation can contribute to exacerbations.

I) Respiratory infections: Viral or bacterial infections of the respiratory tract can cause inflammation and worsen COPD symptoms.

Although there has been ongoing research, there have not been any significant advancements in disease-modifying treatment for COPD lately. Smoking cessation is the only intervention known to change the course of the disease and increase the survival rate [23].

## Review

Pharmacological treatments

Pharmacological treatment for COPD is intended to improve exercise tolerance and general health status and lessen the burden of symptoms and the frequency and severity of exacerbations [11]. Bronchodilators, systemic corticosteroids, and antibiotics are the primary treatment options used in the management plan for acute exacerbations of COPD in the hospital setting [11].

Bronchodilators

Bronchodilators are considered one of the mainstays in treating COPD at all levels of severity [24]. They include beta-2 agonists and antimuscarinic drugs.

Beta-2 Agonists

Beta-2 agonists improve symptoms of COPD exacerbation and FEV1 [25] by activating beta-2 adrenergic receptors, which relax the smooth muscle of the airways, thus raising cyclic adenosine monophosphate (cAMP) and functionally opposing bronchoconstriction [11]. Beta-2 agonists are of two types: short-acting beta-2 agonists (SABAs) and long-acting beta-2 agonists (LABAs). The short-acting are salbutamol (albuterol), levalbuterol, terbutaline, and fenoterol [11]. Long-acting beta-2 agonists significantly reduce the severity of dyspnea, rate of exacerbation, and number of hospitalizations. However, these medicines have no impact on mortality or decline in lung function [11, 26]. The drugs are further classified according to their duration of action and daily dosage. Some are used twice a day, which includes formoterol and salmeterol [26], while indacaterol, oladaterol, and vilanterol are required once a day as they have a duration of action of 24 hours [11, 27-29]. In addition to the clinical benefits, beta-2 agonists possess several harmful effects, too, such as sinus tachycardia, palpitations, somatic tremors, variations in blood pressure, hypokalemia, and increased oxygen consumption [11, 30].

Antimuscarinics

Antimuscarinic drugs cause bronchodilation by inhibiting the acetylcholine effects on muscarinic 3 (M3) muscarinic receptors in smooth airway muscles [11, 31]. Ipratropium and oxitropium are two examples of short-acting antimuscarinics (SAMAs) that additionally block the inhibitory neuronal receptor M2, potentially contributing to vagally induced bronchoconstriction [11, 32]. Tiotropium, glycopyrronium bromide, aclidinium, and umeclidinium are a few examples of long-acting muscarinic antagonists (LAMAs), which prolong the duration of the bronchodilator effect by prolonged binding to M3 muscarinic receptors and quicker dissociation from M2 muscarinic receptors [11, 31]. Unlike beta-2 agonists, these drugs have fewer side effects, which are not severe and are similar to all drugs in this class. It includes dry mouth, urinary symptoms, and a bitter and metallic taste [11].

**Figure 2:**
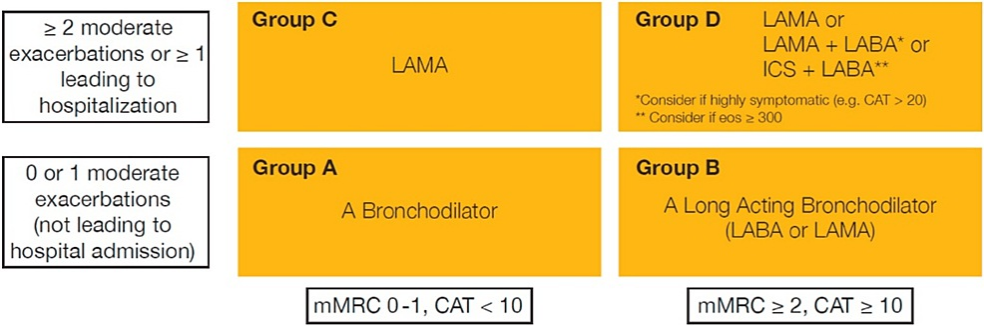
Initial pharmacologic treatment of COPD Reproduced with permission under the Creative Commons Attribution License. CAT: COPD assessment test; ICS: inhaled corticosteroid; LABA: long-acting β2-agonist; LAMA: long-acting muscarinic antagonist; mMRC: modified Medical Research Council dyspnea scale [33]

The model showing the treatment strategy according to symptoms and severity is given in Figure 2.

In the initial management of COPD exacerbations, SABAs with or without SAMA are highly preferred, regardless of the inadequacy of high-quality evidence from randomized controlled trials [30, 34]. Moreover, as per the GOLD 2023 report, no clinical evidence determines the advantageous role of LABAs with or without LAMAs in acute exacerbations of COPD. However, it is recommended to start these drugs during exacerbations or as soon as possible before hospital discharge [11]. Dual LABA therapy and LAMA are highly preferred in moderate-to-severe exacerbations or patients requiring hospitalization. It is also evident from a Cochrane systematic review and network meta-analysis that the combination therapy of LABA in conjugation with LAMA was considered the top-ranked therapy for reducing the exacerbation of COPD [11, 35].

Beta Blockers

The effects of beta (β)-receptor activation are countered by beta blockers, which bind specifically to beta receptors and prevent beta-receptor agonists from binding to those receptors. Beta blockers are divided into cardio-selective and non-selective blockers as per different receptor subtypes. Non-selective β-blockers, including propranolol and carvedilol, act on both β-1 and β-2 receptors [36], whereas cardioselective β-1 blockers such as atenolol, bisoprolol, and metoprolol have a 20-fold higher affinity for β-1 receptors as compared to β-2 receptors, which makes them less susceptible to causing bronchoconstriction [36, 37]. When respiratory conditions are present, review articles and practice recommendations demonstrate a clinical reluctance to administer selective beta-1 blockers; however, a Cochrane review found that beta-1 blockers did not impair beta agonist responsiveness or the ability to treat COPD [38, 39]. Different observational studies have shown evidence that suggests that β blockers cause a marked reduction in hospital admissions, mortality, hospital visits, especially in the emergency room (ER), and COPD exacerbations in patients with COPD with or without cardiovascular disease (CVD) [37, 40-43]. Despite various studies showing the positive effects of β-blockers, especially cardio selective β1 blockers, on COPD exacerbation and mortality, the recent GOLD report 2023 recommended that selective β1 blockers should only be used in patients having COPD concomitantly with a CVD [11, 44]. The summarized results of various studies are shown in Table 1.

**Table 1:** Summary of main findings to assess the effects of beta blockers on patients with COPD Reproduced under the terms of the Creative Commons Attribution License. NA: not available; COPD: chronic obstructive pulmonary disease; CHF: chronic heart failure; HF: heart failure; MI: myocardial infarction; CAD: coronary atherosclerotic heart disease; AECOPD: acute exacerbation of chronic obstructive pulmonary disease; GOLD: Global Initiative for Chronic Obstructive Lung Disease; BB: beta blockers; CI: confidence interval; IRR: incidence rate ratio; OR: odds ratio; HR: hazard ratio [36]

Author, Year	Type of Study	Study Population	Sample Size	GOLD Rating	Drug Types	Follow-Up Time	Exacerbation Risk	Mortality Risk	Pulmonary Function
Duﬀy, 2017 [39]	Retrospective observational	COPD (with or without cardiac complications)	1219	III or IV	NA*	180 days	No beneﬁcial or detrimental eﬀect	NA	NA
Short, 2011 [40]	Retrospective observational	COPD	2712	I-IV	Non-selectiveand cardioselective	4.35years	Decreased	Decreased adjusted HR = 0.78 (95% CI: 0.67 –0.92)	No detrimental eﬀect
Bhatt, 2016 [43]	Prospective observational	COPD	3464	II-IV	NA	2.1 years	Decreased IRR (total AECOPD) =0.73 (95% CI: 0.60– 0.90), IRR (severe exacerbations) = 0.67 (95% CI: 0.48 –0.93)	No beneﬁcial or detrimental eﬀect	NA
Dransﬁeld, 2019 [44]	Prospective, Randomized trial	COPD	532	≥II	Metoprolol	NA	NA	Increased HR = 1.91 (95% CI: 1.29 to 2.83)	NA
Puente-Maestu L, 2014 [45]	Retrospective observational	COPD with CHF or CAD	256	I-IV	NA	NA	Decreased adjusted OR = 0.27 (95% CI: 0.15 – 0.50)	NA	NA
Neef, 2017 [46]	Retrospective observational	AECOPD	36	I-IV	Non-selective and cardioselective	NA	No beneﬁcial or detrimental eﬀect	NA	NA
Sessa, 2018 [47]	Retrospective observational	COPD + HF	14,339	NA	Non-selective and cardioselective	60 days	Increased adjusted HR = 1.61 (95% CI: 1.52 – 1.70)	NA	NA
Rutten, 2010 [48]	Retrospective observational	COPD	2230	NA	Non-selective and cardioselective	7.2 years	Decreased adjusted HR =0.71 (95% CI: 0.60– 0.83)	Decreased adjusted HR = 0.68 (95% CI: 0.56 –0.83)	NA
Quint, 2013 [49]	Retrospective observational	COPD + MI	1063	NA	NA	2.9 years	NA	Decreased adjusted HR (BB started during hospital admission) = 0.50 (95% CI: 0.36 to0.69). Adjusted HR (BB started before hospital admission) = 0.59 (95%CI: 0.44 to 0.79)	NA
Liao, 2017 [50]	Retrospective observational	COPD + HF	1872	NA	Non-selective and cardioselective	90 days	No beneﬁcial or detrimental eﬀect	Decreased (high-dosebisoprolol) adjusted HR = 0.51 (95% CI:0.29 to 0.89)	NA
Kubota, 2015 [51]	Retrospective observational	COPD + CHF	132	NA	Non-selective and cardioselective	33.9 months	Decreased (bisoprolol) unadjusted HR =0.38 (95% CI:0.15 – 0.98)	Decreased (univariate analysis) unadjusted HR = 0.41 (95% CI:0.17 –0.99); no beneﬁcial or detrimental eﬀect (multivariate analysis)	NA
Ekstrom, 2013 [52]	Prospective observational	Oxygen-dependent COPD	2249	NA	NA	Four years	NA	Increased adjusted HR= 1.19 (95% CI: 1.04 to1.37)	NA
Oda, 2017 [53]	Retrospective observational	COPD	103	I-IV	Non-selective and cardioselective	4.1 ± 2.5 years	No signiﬁcant change	No signiﬁcant change	No signiﬁcant change
Maltais, 2018 [54]	Retrospective observational	COPD	557	II-IV	NA	24 or 52 weeks	No signiﬁcant change	No beneﬁcial or detrimental eﬀect	Improved from baseline

Table 2 below comprehensively sheds light on studies reporting the use of systemic corticosteroids to manage COPD exacerbations.

**Table 2:** Use of systemic corticosteroids in the management of COPD exacerbations mg: milligrams; COPD: chronic obstructive pulmonary disease; N/A: not available; FEV1: forced expiratory volume in 1 second

Author	Year	Type of Study	Drug	Dose and Route of Administration	Course of treatment	Clinical Use and Benefit	Effect on Hospital Stay	Effect of Mortality	Adverse Effects
Ian D Pavord et al. [55]	2022	Review article	Prednisone	40mg, oral	Five days	COPD exacerbations in hospitalized patients	Decrease in hospital stay	No significant effect on mortality	Hyperglycemia
Fekri Abroug et al. [56]	2014	Meta-analysis	Methylprednisolone or prednisone.	Oral or Intravenous	Five to 15 days	COPD exacerbations and overall improvement in overall lung function	Decrease in hospital stay	No effect on mortality rate	Increased episodes of hyperglycemia
Mona Bafadhel et al. [57]	2014	Randomized controlled trial	Prednisolone	30–40 mg prednisolone	10–14 days	Patients with COPD exacerbations and those having a >2% eosinophil count	Overall prompt response but no evidence about hospital stay duration.	N/A	N/A
Yelda Ceviker et al. [58]	2014	Randomized parallel-group study	Methylprednisolone	32 mg/day	Seven days	Betterment in lung function, symptoms, and oxygenation in patients with COPD exacerbations	N/A	N/A	N/A
Hammad Qureshi et al. [59]	2014	Randomized control trial	Prednisolone and methylprednisolone	30–40 mg oral route or 125 mg intravenous	Four times daily for three days.	Rapid improvement in FEV1 and hypoxemia	No effect. Decrease in Hospital stay	No effect	Insomnia, increased appetite, weight gain, hyperglycemia, and secondary infections
Claus F Vogelmeier [60]	2014	Review article	Prednisolone	40 mg oral	Five days	Improvement in the function of the lung, breathing, and hypoxemia	Decrease in hospital stay	N/A	Hyperglycemia with chronic use
Ernesto Crisafulli et al. [61]	2018	Narrative review	Prednisolone and methylprednisolone	500 mg for 3 days followed by oral prednisone 60 mg for 4 days.	14 days	Betterment in oxygenation, breathing, FEV1, and recurrence risk. A short duration has a better outcome than a longer duration of the drug. Improvement in FEV1 and reduction in hospital stay. (There is no evidence of benefit of the route of administration as both have the same adverse effects. Also, low oral doses, compared to high intravenous doses, should be preferred because there is no associated benefit with the latter. However, a decrease in adverse effects was seen with low oral doses). In other words, both routes have shown the same efficacy.	Decrease in hospital stay	N/A	Osteoporosis, hyperglycemia, and muscle weakness
Pedro J. Marcos et al. [62]	2017	Observational cohort study	Prednisone	40 mg/day	Five-day course	Less severe exacerbations that do not require hospitalization	The study concluded that an increase in the duration of treatment increases the stay in the hospital.	N/A	N/A
A.J. Reis et al. [63]	2018	Review article	Prednisolone and prednisone	30 mg oral and 40 mg oral, respectively.	Seven days and five days, respectively.	No additional benefit is noted for a longer therapy. Also, this dose decreases the time of recovery, improvement in lung function, improvement in oxygenation, decreases the risk of recurrence, and decreases the chances of treatment failure.	Decrease in hospital stay	N/A	N/A

Table 3 outlines studies from the literature that have used antibiotics to treat COPD exacerbations.

**Table 3:** Use of antibiotics in COPD exacerbations COPD: chronic obstructive pulmonary disease; N/A: not available; mg: miligrams

Author	Year	Type Of Study	Drug	Dose and Route of Administration	Course of treatment	Clinical Use	Effect on Hospital Stay / Re-admission	Effect of Mortality	Adverse Effects
Mihaela S. Stefan MD et al. [64]	2012	Retrospective cohort study	Quinolone and macrolide + cephalosporin macrolide monotherapy	N/A	Two days	Acute exacerbations of COPD	13% decrease in readmission	40% decrease in mortality	*Clostridium difficile* colitis
Hammad Qureshi et al. [59]	2014	Randomized controlled trial.	Macrolides & B-lactam agents (should be combined with B-lactam inhibitors), Fluoroquinolones, and Tobramycin	Oral and inhaled	< Five days	For normal exacerbations, severe exacerbations, history of antibiotic use, and history of oral corticosteroid use. Efficient against resistant strains of *Haemophilus influenzae *and *Streptococcus pneumoniae*. Severe COPD with colonization of *Pseudomonas*. Decreases sputum inflammatory markers and colonies of bacteria.	N/A	N/A	N/A
MeiLan K. Han et al. [65]	2014	Randomized controlled trial	Azithromycin	250 mg	Daily for one year	Most effective in preventing acute exacerbations in patients requiring systemic steroids and antibiotics. The most significant advantage was seen in older patients. No benefit for current smokers was seen.	N/A	N/A	Minor hearing loss and nasopharyngeal colonization with azithromycin-resistant organisms.
Patricia van Velzen MD et al. [66]	2017	Randomized double-blind placebo-controlled trial	Doxycycline	Oral 100 mg daily and 200 mg on the first day	Seven-day course	COPD exacerbations in the outpatient department	No significant effect	No significant effect	N/A
A.J. Reis et al. [63]	2018	Narrative review.	Aminopenicillin with clavulanic acid, a macrolide, or a tetracycline.	N/A	Five to seven days	COPD exacerbations	N/A	N/A	Long-term use of macrolides may lead to the risk of developing bacterial resistance.

Non-invasive and invasive ventilation

Ventilation refers to providing oxygen (O_2_) and removing carbon dioxide (CO_2_). Ventilation is required in patients to halt the progression of cardiorespiratory compromise, which can either manifest as a rising respiratory rate, an asynchronous pattern of respiration, an alteration in mental status and consciousness level, persistent oxygen desaturation despite increasing oxygen concentration, hypercapnia and respiratory acidosis, or circulatory problems, including hypotension and atrial dysrhythmias [67]. The modern ventilator combines air with variable concentrations of O_2_ under high pressure, providing inspiration and expiration. Three operator-adjusted factors constitute a ‘breath’ [67]. They are :

A) Trigger: A specific rate (ventilator-initiated/ mandatory breaths) at which the ventilator will deliver the breath or alteration in pressure or flow in the ventilator occurring due to conscious breathing by the patient (patient-initiated/spontaneous breaths) [67].

B) Target: Air flowing into the lungs at a fixed rate (volume control) or pressure (pressure control; pressure support; bi-level) [67].

C) Termination: The halting of inspiration and the beginning of expiration are conveyed via a signal to the ventilator. This process can be volume controlled (volume cycled), time held (time cycled: pressure control/bi-level), or flow controlled (decreasing inspiratory flow to a preset level: flow cycled: pressure support) [67].

There are two significant divisions in ventilation practices: non-invasive and invasive.

Non-invasive Ventilation (NIV)

**Figure 3:**
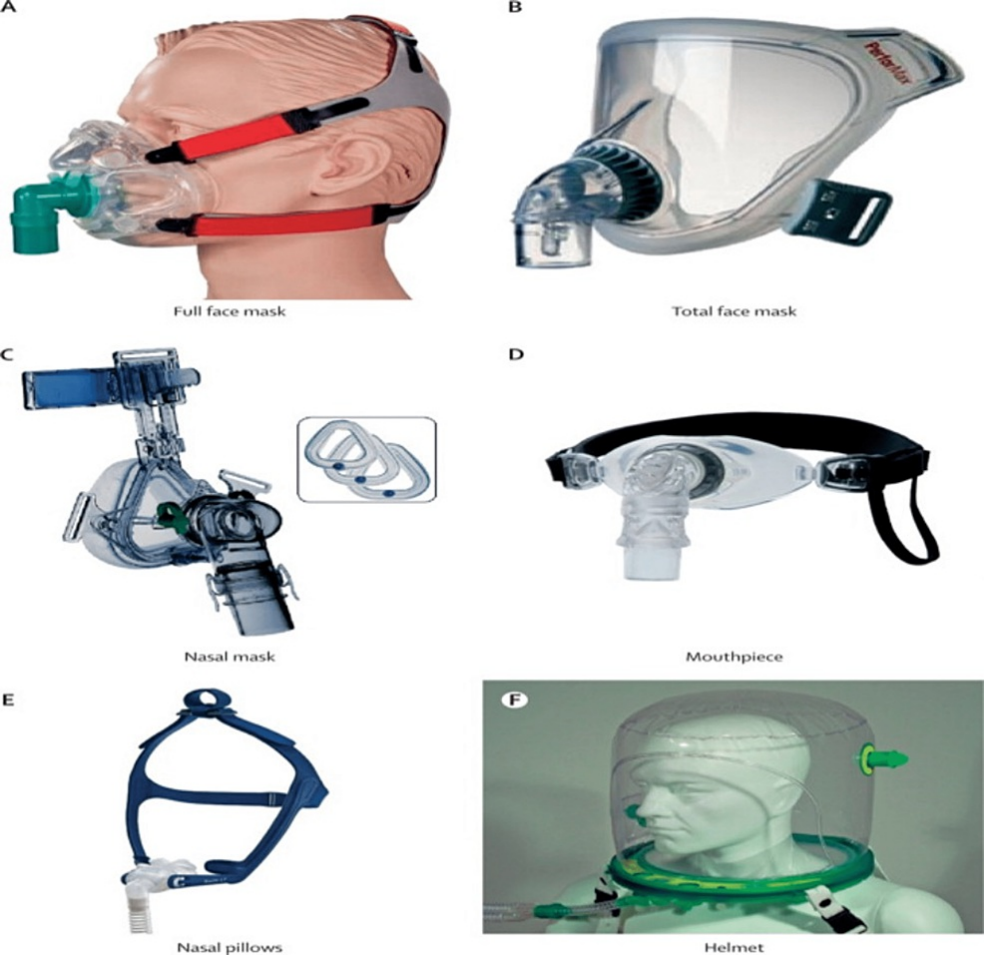
Different types of NIV interfaces Reproduced under the terms of the Creative Commons Attribution license. NIV: non-invasive ventilation [67]

As the name suggests, NIV means providing respiratory assistance while avoiding tracheal intubation. This allows us to prevent harm from complications arising from invasive ventilation, including the requirement of sedation, which poses a risk for hemodynamic compromise as well as delirium, hospital-acquired infections, etc. [68]. Figure 3 shows different types of NIV interfaces.

Recent guidelines from the American Thoracic Society (ATS) in 2020 recommend the following uses of noninvasive positive pressure (NPPV) in acute respiratory failure (ARF) [69]. Bilevel-positive airway pressure (BiPAP) can be used for COPD exacerbation with acute or acute chronic respiratory acidosis with a pH</=7.35 [69]. Bilevel-positive airway pressure can be used in order to avoid invasive ventilation in relatively stable patients who are not deteriorating at the moment [69]. Bilevel-positive airway pressure, or continuous positive airway pressure (CPAP), can be used in cardiogenic pulmonary edema [69]. Guidelines also provide conditional recommendations in ARF [69], such as early use of NIV for immunocompromised patients with ARF, postoperative ARF, use in patients with dyspnea suffering from terminal conditions like cancer as a means to provide palliative care, chest trauma patients with ARF, and in order to protect high-risk patients from post-extubation respiratory failure.

Invasive Ventilation 

It requires access to the trachea, usually via an endotracheal tube. Although this method is associated with numerous complications, its indications are extremely rigid. Table 4 shows indications for the use of invasive ventilation.

**Table 4:** Indications for the use of invasive ventilation Reproduced under the terms of the Common Creative Attribution license. NIV: non-invasive mechanical ventilation [70]

Treatment Option of Choice	Indications
Invasive ventilation	Unable to tolerate NIV or NIV failure
	Respiratory or cardiac arrest
	Respiratory pauses with loss of consciousness or gasping for air
	Diminished consciousness, psychomotor agitation inadequately controlled by sedation
	Massive aspiration
	Persistent inability to remove respiratory secretions
	Heart rate <50 min⁻¹ with loss of alertness
	Severe hemodynamic instability without response to fluids and vasoactive drugs
	Severe ventricular arrhythmias
	Life-threatening hypoxemia in patients unable to tolerate NIV

Different Modes of Ventilation 

Mechanical ventilation mode should be selected based on the safety profile, which can be achieved by optimizing the efficiency of ventilation-perfusion matching and pressure-volume mechanics of the lungs [71]. Intensive care units (ICUs) use assist-control (AC) mode, which is one of the most common methods of mechanical ventilation [72]. In the AC mode, volume cycling is an important feature; this means that a particular volume will always be delivered, with lung compliance playing an important role in determining the pressure being generated. Low plateau pressures are generated by lungs with high compliance; likewise, very high pressures are generated by lungs with low compliance (i.e., stiff lungs), as seen in patients with acute respiratory distress syndrome (ARDS), pulmonary edema, fibrosis, and pneumonia. This knowledge allows us to make changes to prevent barotrauma [73, 74].

Benefits of the AC Mode [73]

The feature of triggering breath as needed leads to enhanced patient comfort. Respiratory alkalosis/acidosis can easily be resolved by the operator seamlessly correcting the CO_2_ levels. This mode requires less breathing work for the patient.

Drawbacks of the AC Mode [73]

Barotrauma remains an important concern in stiff lungs as this system is volume-cycled, but it could be avoided by regularly checking the plateau pressures. Breath stacking leading to auto-positive end-expiratory pressure (PEEP) can occur if a patient does not have enough time for exhalation, as in the case of tachypnea. Not exhaling the entire volume leads to a progressive accumulation of air in the patient’s lungs. Consequently, the plateau and intrathoracic pressures are increased, interfering with the venous return and causing hypotension. This is managed by taking the patient off the ventilator to allow for proper exhalation and readjusting the settings for further use.

Voluntary control to initiate breaths can lead to respiratory alkalosis due to hyperventilation. Adequate sedation resolves this problem. New ventilator modes are designed to counteract some of the drawbacks of AC mode [74,75]:

Neurally adjusted ventilator assist (NAVA): The timing and intensity of the diaphragm measured via the sensors are matched with the timing and intensity of the ventilator. This allows us to fulfill the ventilator demand and avoid barotrauma [76-78].

Adaptive support ventilation (ASV): Reaching a target minute ventilation via automatic adaptation of inspiratory pressure and respiratory rate. This mode has the added benefit of requiring minimum effort on the part of the patient [79].

Airway pressure release ventilation (APRV): In this pressure-cycled mode, the air is delivered at a particular pressure for a specified amount of time, then the lung is allowed to deflate for a short period, which helps in preventing alveolar damage. This mode is particularly useful when AC cannot be used safely because of constantly elevated plateau pressures and poor oxygenation [80].

Clinical outcomes 

A randomized, prospective study was conducted in the ICU of the pulmonology unit of a civil hospital in Jamshoro, Pakistan. The study enrolled patients with diagnosed cases of COPD who were admitted to the ICU due to acute respiratory failure during the period from January to December 2018 [81]. Patients with both hypercapnic (partial pressure of arterial carbon dioxide (PaCO_2_) > 50 mmHg; pH < 7.30) and hypoxemic (partial pressure of oxygen (PaO_2_) <60 mmHg) respiratory failure [82] were included. Treating pulmonologists and critical care specialists decided whether to give non-invasive positive pressure ventilation (NIPPV) or invasive positive pressure ventilation (IPPV) on the basis of biochemical impairments and clinical findings. Table 5 summarizes the criteria used to select and exclude patients for NIPPV and the indications for IPPV.

**Table 5:** Selection and exclusion criteria for NIPPV and indications of IPPV Reproduced under the terms of the Creative Commons Attribution License. IPPV: invasive positive pressure ventilation; NIPPV: non-invasive positive pressure ventilation; PaO_2_: partial pressure of oxygen; PaCO_2_: partial pressure of carbon dioxide [83]

Criteria	Severity
Selection criteria for NIPPV (at least two should be present)	Moderate to severe dyspnea, accompanied by the use of accessory muscles and paradoxical abdominal motion
	Moderate to severe acidosis (pH 7.30–7.35) and hypercapnia (PaCO_2_ 45–60 mmHg)
	Respiratory frequency > 25 breaths/minute
	Moderate to severe hypoxemia (PaO_2_ < 60 and PaCO_2_ <45 mmHg)
Exclusion criteria for NIPPV (any of these may be present)	Respiratory arrest
	Cardiovascular instability (hypotension, arrhythmias, myocardial infarction)
	Somnolence, impaired mental status, uncooperative patient
	High aspiration risk; viscous or copious secretions
	Extreme obesity
	Recent facial or gastroesophageal surgery
	Craniofacial trauma, fixed nasopharyngeal abnormalities
Indications of IPPV	Severe dyspnea accompanied by the use of accessory muscles and paradoxical abdominal motion
	Respiratory frequency > 35 breaths/min
	Life-threatening hypoxemia (PaO_2_ 40 mmHg)
	Severe acidosis (pH < 7.25) and hypercapnia (PaCO_2_ > 60 mmHg)
	Respiratory arrest
	Somnolence and impaired mental status
	Cardiovascular complications (hypotension, shock, heart failure)
	Other complications (metabolic abnormalities, sepsis, pneumonia, pulmonary embolism, barotrauma, massive pleural effusion)
	NIPPV failure

Devi et al. conducted a study that evaluated the following outcomes: duration of ventilation, length of ICU stay, length of hospital stay, mortality within the ICU or post-ICU within the hospital, and failure of NIPPV, requiring the need to start IPPV. Table 6 presents data indicating that within the NIPPV group, there was no statistically significant reduction in PaCO_2_ after 24 hours of ventilation (p=0.28).

**Table 6:** Comparison of demographic and clinical characteristics of patients on invasive and non-invasive positive pressure ventilation Reproduced under the terms of the Creative Commons Attribution license. *For both groups (NIPPV and IPPV), values at baseline were compared with those after 24 hours of ventilation. BP: blood pressure; IPPV: invasive positive pressure ventilation; NIPPV: non-invasive positive pressure ventilation; PaO_2_: partial pressure of oxygen; PaCO_2_: partial pressure of carbon dioxide; SD: standard deviation [81]

Patients Characteristics	At Baseline			At 24 Hours of Ventilation			P-value Within Groups*	
	NIPPV (n=30)	IPPV (n=30)	P-value	NIPPV (n=30)	IPPV (n=30)	P-value	NIPPV (n=30)	IPPV (n=30)
Gender				Not applicable				
Male n (%)	18 (60%)	21 (70%)	0.41					
Female n (%)	12 (40%)	9 (30%)						
Age (years)	65.4 ± 11.8	73.6 ± 13.2	0.01					
PaCO_2_ (mmHg)	63.2 ± 21.5	78.1 ± 20.2	0.007	57.2 ± 21.5	69.1 ± 20.2	0.03	0.28	0.08
PaO_2 _(mmHg)	61.3 ± 18.9	60.2 ± 21.4	0.83	74.6 ± 19.3	65.7 ± 13.4	0.04	0.009	0.23
Respiratory rate per minute	27.4 ± 3.1	29.7 ± 4.8	0.03	25.2 ± 3.1	27.9 ± 6.2	0.03	0.008	0.21
Heart rate per minute	113.4 ± 9.7	121.1 ± 11.3	0.006	103.2 ± 6.1	118.4 ± 9.5	<0.0001	<0.0001	0.32
Systolic BP (mmHg)	146.2 ± 17.3	137.9 ± 13.4	0.04	138.1 ± 10.7	130.2 ± 7.5	0.001	0.03	0.008
Diastolic BP (mmHg)	68.5 ± 15.6	86.1 ± 18.1	0.0002	84.2 ± 11.8	92.4 ± 15.3	0.02	<0.0001	0.15

However, in the IPPV group, this reduction was found to be significant (p=0.08). Regarding PaO_2_ improvement with 24 hours of ventilation, the NIPPV group exhibited a statistically significant improvement (p=0.009), while such improvement was not significant in the IPPV group (p=0.23) [81]. The NIPPV group demonstrated a statistically significant improvement in respiratory rate (p=0.008), whereas the IPPV group failed to demonstrate any significant improvement [81]. The NIPPV group showed a noteworthy and statistically significant improvement in heart rate (p<0.0001). Both groups experienced a significant reduction in systolic blood pressure, while diastolic blood pressure reduction was observed solely in the NIPPV group. All demographic and clinical profile variables can be found in Table 6 [81]. PaCO_2_, PaO_2_, respiratory rate, heart rate, and blood pressure of the NIPPV and IPPV groups at baseline (before any medical intervention) and after 24 hours of ventilation are also shown and compared in Table 6 [82].

The patient outcomes were evaluated and compared between the two groups. The group that received NIPPV demonstrated significantly reduced durations of ventilation, ICU stays, and post-ICU hospitalization. The mortality rate within the ICU was 13.3% for the NIPPV group, while it was 40% for the group receiving IPPV (p=0.01) [81]. In comparison to the NIPPV group, which received ICU care, the mortality rate was 6.7% versus 16.% for the IPPV group. However, it was discovered that there was no statistically significant difference between these groups. Within the first 24 hours of being hospitalized, no reported deaths occurred. A transfer to invasive ventilation was necessary for 20% (n=6) of the NIPPV patients who had unsuccessful non-invasive ventilation. Six of these patients underwent ICU treatment; three (or 50%) of them died there; the other three recovered and were released. After extubation, no cases of ICU readmission or re-ventilation requirements were noticed [81]. When compared to IPPV, NIPPV has been shown to significantly improve patient outcomes, even in ARF secondary to causes other than COPD [81]. In a meta-analysis of 13 observational studies involving immunocompromised patients admitted to the ICU with ARF, NIPPV significantly decreased in-hospital mortality (odds ratio (OR): 0.43, p-value = 0.007) and 30-day mortality (OR: 0.34, p-value) [84]. Non-invasive ventilation was used in a different meta-analysis with more than 2000 patients who had orders to do without intubation (DNI) and only use comfort measures. When patients with DNI were discharged, the combined survival rate was 56% and 32% after a year. The hospital survival rate with NIPPV was 68% in COPD patients with DNI orders [85].

Smoking Cessation 

**Figure 4:**
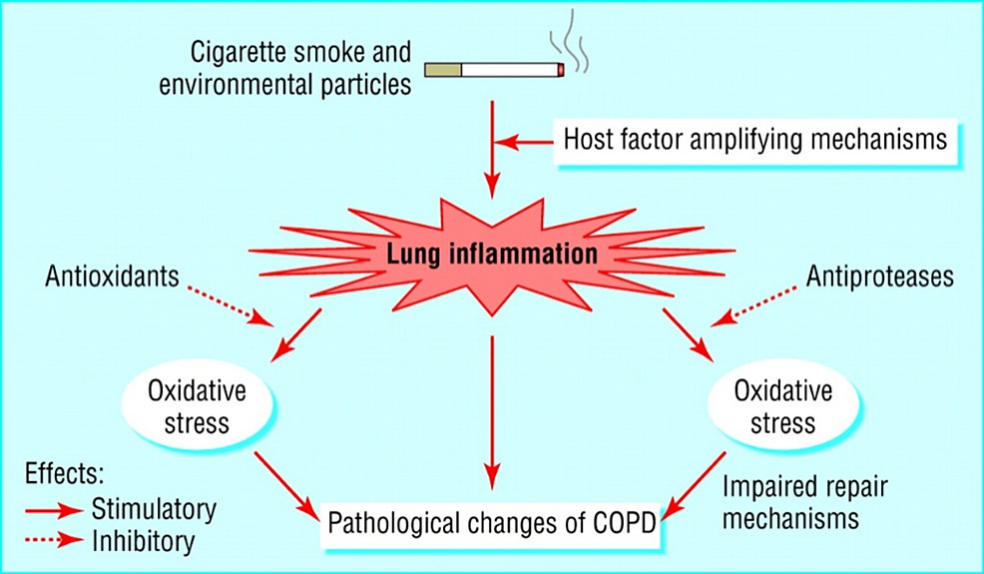
The pathogenesis of COPD Reproduced under the terms of the Creative Commons Attribution license. Dashed bars represent inhibitory effects. COPD: chronic obstructive pulmonary disease [86]

The hallmarks of COPD are increased resistance to the airflow of the small conducting airways, increased compliance, or elastic deformation of the lungs, trapping of air leading to irreversible airflow obstruction, and a hyperinflammatory response, particularly in the small airways of the lungs. This hyperinflammatory response, or abnormal immune response, is an adaptive defense mechanism to long-term exposure to noxious particles and gases like cigarette smoke, and this leads to hypersecretion (chronic bronchitis), destruction of lung tissue (emphysema), and impairment of normal defense mechanisms, which causes further inflammation and fibrosis (bronchiolitis) [86]. Figure 4 summarizes the pathogenesis of COPD.

In all smokers, inflammation in the small airways is evident and gets further amplified in smokers with COPD, causing structural changes that increase with disease severity and even persist after cessation of smoking [86]. Pathological processes in COPD, such as inflammation, the imbalance between proteases and anti-proteases, and oxidative stress in the lung [86], are interconnected and related to exposure to cigarette smoke. This explains why smoking is the main aggravating factor in COPD and why the cessation of smoking in COPD patients is of utmost importance. Smoking cessation is the most effective method to limit the progression of the disease, leading to positive outcomes such as reduced dyspnea, decreased frequency of exacerbations and hospitalizations, slowed-down decline in lung function, and decreased mortality [87].

Strategies for smoking cessation could either be pharmacological or non-pharmacological approaches, but first, the healthcare professional needs to identify whether the patient is an active or passive smoker. Together with a thorough medical assessment, the healthcare professional has to follow up until the goal is achieved. The strategy termed ‘brief advice’ motivates patients to quit smoking and to seek treatment through a single intervention. This has shown a higher frequency of quit attempts than without interventions, and studies show that behavioral support shows better results than offering medication [88]. A model on how to give brief advice has been proposed, which includes five steps known as "The 5 A's Approach" (Ask, Assess, Advice, Assist, Arrange) [88, 89] and is as follows:

1) Ask: Inquire about tobacco use and smoking status.

2) Assess: Evaluate the patient’s willingness to quit smoking and assess their nicotine dependence.

3) Advice: Strongly and clearly counsel patients into quitting smoking.

4) Assist: Set up a plan to begin quitting, and if the patient is nicotine dependent, pharmacological approaches have to be explained. Motivate the patient and provide assistance in the forms needed by the patient.

5) Arrange: Follow-ups have to be arranged during the week of the quit day and encourage the patient to continue the progress. Monitor the patient and review if there are any problems.

Studies suggest that light smokers (those who smoke less than 10 cigarettes per day) do not require pharmacotherapy for smoking cessation, but a small percentage of patients seek medications to quit. Studies also show that more than 50% of light smokers failed in their quitting attempts, suggesting they need pharmacological or psychological support since even smoking less than 10 cigarettes per day still predisposes them to COPD and other tobacco consumption-related diseases [88]. Non-pharmacological approaches include one-to-one counseling, individual or group cognitive behavioral therapy (CBT), counseling via telephone, and mobile apps designed with programs to help smoking cessation. Studies suggest that individual behavioral interventions play a significant role in achieving smoking cessation goals [88].

Further Pharmacological Approaches for Smoking Cessation

Varenicline: Varenicline is the most effective drug of choice for smoking cessation, but it is associated with adverse effects that result in early discontinuation of the drug, leading to failure of the goal [90]. Compared to nicotine replacement therapy, varenicline is not associated with an increased risk of depression and can be prescribed to COPD patients [88].

Nicotine replacement therapy (NRT): The aim is to reduce nicotine withdrawal symptoms, and from studies, it has been found that using two types of NRT is more effective with increased rates of smoking cessation than using one. The combination of a rapid delivery form of NRT like chewing gum, lozenges, nasal spray, and inhalers, with a slow-releasing form like a nicotine patch is more effective [91].

Bupropion: It is the first non-nicotinic drug approved by the Food and Drug Administration (FDA), has shown a higher abstinence rate in the long term, and therefore it is a first-line drug for smoking cessation [88]. When comparing the quitting rate, studies show that it is approximately similar for varenicline and NRT but less for bupropion compared to varenicline and NRT [87].

Cytisine: It is a plant-based alkaloid, and its mechanism of action is similar to that of varenicline as it is a selective partial agonist of nicotinic acetylcholine receptors. A study comparing cytisine and varenicline reported that quitline referrals and retention rates were similar. However, the reported adverse effects for cytisine were less frequent than those for varenicline, and the most frequently reported adverse effects, which were abnormal dreams and nausea, were more frequently reported for varenicline [90].

Vaccine: The mechanism of action will be to produce antibodies to bind to nicotine and reduce its effect. A vaccine is indicated for patients who have failed at many different methods of quitting. Vaccine development is still under study, and modifications that are designed to improve the affinity of serum antibodies are still being tested [88].

Electronic cigarettes: The smoking cessation rates when using electronic cigarettes with nicotine were increased when compared to those with placebo or nicotine patches, but due to the inclusion of other chemicals like lung irritants, less knowledge about the long-term effects of electronic cigarettes, and no FDA approval, it is less effective as a treatment for smoking cessation [91].

Combined treatment with medications: Trials have demonstrated that combined treatment methods increase the probability of successful outcomes. This can either be a psychological intervention with medication or a combined pharmacological treatment [88]. Clinical trials carried out to assess effective smoking cessation interventions in patients with COPD found that gradual smoking cessation gave better positive results than abrupt cessation [92]. However, in studies carried out to assess whether the commitment to smoking cessation has an impact on smoking cessation efficacy, it was found that adherence or commitment to smoking cessation treatment significantly affects and improves smoking cessation efficacy [93].

Oxygen prescription

Oxygen therapy systems used in COPD can be categorized as low-flow and high-flow devices [94].

Low-flow devices include [94]:

1) Nasal cannula: This is an old method that involves the use of nasal cannula prongs of various sizes and types. It succeeded in using an elastic band that could fit over the patient’s ears.

2) Simple mask: This is used when a higher fraction of inspired oxygen (FiO_2_) needs to be given to the patient. It fits over the bridge of the nose and is kept in place using an elastic band that goes over the head.

3) Non-rebreather: It is comparable to a mask but also includes a reservoir bag connected to the mask by a valve.

4) Venturi mask: This type of face mask allows a certain controlled amount of previously determined FiO_2_.

Modern oxygen delivery methods incorporate therapies like CPAP and BPAP into noninvasive ventilation (NIV). Continuous positive airway pressure (CPAP) therapy is a method used to provide a continuous level of PEEP during both inspiration and expiration, thus increasing the patient's FRC [94]. Bilevel positive airway pressure (BPAP) therapy uses a pressure cycling mode for delivering NIV [94].

High-flow devices include [94]:

1) Optiflow^TM^(Fisher & Paykel Healthcare): It allows the mixing of 100% oxygen with room air and creates the necessary FiO_2_ while providing a high flow rate of humidified gas. It is known to reduce the risk of endotracheal intubation in COPD patients with acute exacerbations (AECOPD) [95].

2) Airvo^TM^ 2 (Fisher & Paykel Healthcare): It is a humidifier with a built-in flow generator that provides high-flow-warmed and humidified respiratory gases to spontaneously breathing patients. This can improve mucociliary clearance of secretions more effectively.

The flow rates for the devices used for oxygen delivery are mentioned in Table 7 as follows:

**Table 7:** Devices used for oxygen delivery and their flow rates FiO_2_: fraction of inspired oxygen

Method of Oxygen Delivery	Flow Rate	FiO_2_	Reservoir Size
Nasal cannula [94]	1-6 L/min	0.22 to 0.24 at 1 L/min, 0.4 at 5-6 L/min	-
Simple mask [94]	5-10 L/min	0.3-0.8 L/min	100-200 mL
Non-rebreather [94]	15 L/min	0.6-0.9 L/min	300-600 mL
Optiflow^TM^ [94, 95]	60 L/min	0.3 L/min	-

Based on the data in Table 8, we can see that NIV and high-flow oxygen delivery methods do not show significant differences in the outcome of treating AECOPD patients, especially the criteria for intubation.

**Table 8:** Outcomes of high-flow rate methods of oxygen delivery and noninvasive ventilation SpO2: oxygen saturation; FiO_2_: fraction of inspired oxygen; AECOPD: acute exacerbations of COPD, GOLD: Global Initiative for Chronic Obstructive Lung Disease; NIV: non-invasive ventilation; PaCO^_2_^:^_ _^particle pressure of carbon dioxide

Author	Year	Study Design	Method of Oxygen Delivery	Inclusion Criteria	Sample Size	SpO_2_	Flow Rate	Gas Temperature	FiO_2_(median)	Criteria for Intubation	Duration of Treatment	Length of Hospital Stay(median)	Readmission Rate	Mortality Rate	Success Rate
Jingen Xia, Sichao Gu, [95]	2022	Randomized controlled trial	High-flow nasal oxygen therapy (Airvo-2™)	AECOPD patients with mild hypercapnia (pH ≥ 7.35 and arterial partial pressure of carbon dioxide > 45 mmHg)	163 (only 158 completed)	90-95%	Initial-30L/min After 72hrs- 35L/min.	31–37 °C	0.32	4 (2.5%)	Every day till flow rate was <20L/min and FiO_2_was <0.3	9.0	At day 90-25 (16.3%)	-	-
Jingen Xia, Sichao Gu, [95]	2022	Randomized controlled trial	Conventional oxygen therapy	AECOPD patients with mild hypercapnia (pH ≥7.35 and arterial partial pressure of carbon dioxide >45 mmHg)	174 (only 172 completed)	90-95%	1-5L/min	-	-	1 (0.6%)	-	8.0	23 (13.5%)	-	-
Andrea Cortegiani [96]	2020	Randomized controlled trial	High-flow nasal oxygen therapy (Optiflow™, Airvo™)	Mild-to-moderate AECOPD	40	88-92%	Initial-60L/min	37°C	-	-	Six hours	-	-	-	PaCO^2 ^reduction after two hours- 6.8 mmHg
Andrea Cortegiani [96]	2020	Randomized controlled trial	NIV- full face or oro-nasal mask	Mild-to-moderate AECOPD	40	88-92%	-	-	-	-	Six hours	-	-	-	PaCO^2 ^reduction after two hours- 9.5 mmHg
Pieter Veenstra [97]	2022	Retrospective cohort study	High-flow nasal oxygen therapy (Airvo™)	AECOPD patients according to GOLD criteria	173	-	30 L/min	31°C	0.25	-	Until relieved from sputum stasis and dyspnea	9.5	-	-	61%
Pervin Hancı [98]	2022	Feasibility Study	High-flow nasal oxygen therapy (Optiflow™)	AECOPD patients with respiratory rate >30/min, O2 sat.<90%, lack of response to initial medical treatment	23	>90%	Initial-40L/min Increased till-60L/min	37°C	0.3	2	Until stable	10.0	-	ICU-4.3% 60-day MR-8.6%	-

But a few points worth mentioning would be that high-flow methods were non-inferior to NIV in decreasing PaCO_2_ within two hours of treatment [96], and the use of high-flow methods in AECOPD with sputum stasis showed good tolerance among these patients [97].

Immunization With the Pneumococcal Vaccine

Of the different types of bacteria and viruses that cause AECOPD, *Streptococcus pneumoniae* is the most significant, and hence the administration of pneumococcal vaccines (pneumococcal polysaccharide vaccine 23 (PPSV23) and pneumococcal conjugate vaccine 13 (PCV13)) is recommended for COPD patients as a prophylactic measure. The significant virulence factor of *Pneumococci *is their capsular polysaccharide, and it is the basis for serotyping and developing vaccines. This capsular polysaccharide has invasive disease potential [99]. In early 2000, the vaccine used for children was the 7-valent pneumococcal conjugate vaccine (PCV7). In 2010, it was replaced by PCV13, which decreased the incidence of invasive pneumococcal infections in children and adults. In 2017, PCV13 was introduced into the official vaccination calendar for children under five years of age, and for adults with high risk, PCV13 is given with a booster of PPSV23 after two months. Generally, PPSV23 is recommended for adults [99]. Table 9 is a summary of three types of pneumococcal vaccines and their efficacy.

**Table 9:** Pneumococcal vaccines and their efficacy CAP: community-acquired pneumonia; PPV-23: pneumococcal polysaccharide vaccine 23; PCV13: pneumococcal conjugate vaccine 13

Author/Year	Study Design	Sample Size	Pneumococcal Vaccine Type	Efficacy of Vaccine			No. of Cases of CAP			Mortality
I Alfageme, 2006 [100]	Randomized clinical trial	298	PPV-23	All patients- 24%	<65 years- 76%	>65 years- -14%	All patients - 25	<65 years - 3	>65 years - 22	50.8 per 1000 per year
Galina L. Ignatova, 2021 [101]	Cohort study	123	PCV13	-			18			-
Galina L. Ignatova, 2021 [101]	Cohort study	32	PPV23	-			8			-

For prophylaxis of community-acquired pneumonia (CAP) in patients with COPD, the effectiveness of PPV is higher for patients with severe airflow obstructions and for those under the age of 65 years [100]. Studies have been conducted to compare the clinical effectiveness of the above-mentioned vaccines and their effect on the health status of patients with COPD and it was found that both PCV13 and PCV23 are effective during the first one to two years after vaccination. But PCV13 showed a higher level of protection for five-year follow-ups than PCV23 where PCV23 ensured protection only within two years. The risk of complications for patients with PCV23 vaccination also increased after vaccination [101]. The existing recommendations for pneumococcal vaccination are that both PCV13 and PCV23 have to be routinely administered in a series to all adults >65 years of age, for adults over 19 years who have an immunocompromized state, functional or anatomic asplenia, cerebrospinal fluid leak or cochlear implants PCV13 is recommended to be routinely administered [102].

Hospital Discharge

Depending on the severity of the exacerbation, a suitable discharge plan should be made after reassessing and reclassifying the patients according to the GOLD criteria. It includes bronchodilation optimization, comorbidity management, anti-pneumococcal vaccination, smoking cessation, and pulmonary rehabilitation [11].

Inhalers Prescribed at Discharge

Inhaled bronchodilators are crucial to symptom management and are frequently given to prevent or lessen symptoms. They include single (LABA or LAMA alone), dual (LABA/LAMA, LABA/inhaled corticosteroids (ICS)), and triple therapy (LABA/LAMA/ICS) [11]. Usually, on discharge after a moderate or severe exacerbation, dual therapy or triple therapy should be prescribed. A combination therapy of ICS along with LABA is more effective in reducing the rate of exacerbation and improving health status and lung function as compared to a single therapy. Alternatively, if the response to dual therapy is not appropriate in patients with severe and further exacerbations, i.e., ≥2/year and a blood eosinophil count of ≥300 cells/microL, triple therapy of LAMA, LABA, and ICS is recommended as it causes a reduction in the number of exacerbations and severity [11]. Recently, the two large randomized controlled trials named InforMing the PAthway of COPD Treatment (IMPACT) [103] and Efficacy and Safety of Triple Therapy in Obstructive Lung Disease (ETHOS) [104] have provided evidence that the fixed-dose inhaled triple combination improves the overall health status and reduces all-cause mortality when compared with the dual therapy of inhaled long-acting bronchodilation [11]. However, ICS should be given cautiously and should not be given alone as it is associated with serious side effects, which are pneumonia, cataracts, oropharyngeal candidiasis, and an increased risk of osteoporosis [11, 105].

Pulmonary Rehabilitation 

Pulmonary rehabilitation is a key component in COPD management in conjunction with pharmacological therapies [106]. Pulmonary rehabilitation programs are usually delivered by multidisciplinary teams in outpatient departments, including exercise training, education, nutritional support, and psychosocial support. It is the most beneficial therapeutic approach for enhancing exercise tolerance, health status, and dyspnea [11, 107]. It improves the overall quality of life and exercise capacity at all levels of COPD severity; however, the evidence is more compelling in individuals with moderate to severe disease [11, 108].

Additionally, it significantly lowers the risk of readmissions in patients who have recently experienced an exacerbation [11]. It has been reported from the studies that post-exacerbation rehabilitation can reduce readmissions over a three-month period [109,110]. Moreover, it helps in reducing anxiety, depression [111], and all-cause mortality [11]. According to a comprehensive analysis of randomized controlled trials, patients who had pulmonary rehabilitation started during their hospital stay or four weeks after discharge had a lower mortality rate than those without rehabilitation [112]. Empirical data from a sizable population-based cohort of 190,000 hospitalized COPD patients, in which starting pulmonary rehabilitation within 90 days of discharge was associated with a statistically significant decrease in mortality, has supported these conclusions [113].

Oxygen Therapy 

Exacerbation of COPD can cause acute hypoxic and hypercapnic respiratory failure. Hypoxemia is due to a worsening of the ventilation-perfusion (V/Q) mismatch [114]. In hospitalized patients with COPD exacerbations, the goal oxygen saturation is 88%-92%, or an arterial oxygen pressure (PaO_2_) of approximately 60-70 mmHg. This goal minimizes the risk of worsening hypercapnia associated with liberal oxygen supplementation [11, 115, 116]. Compared with a higher target of oxygen saturation, a target of 88%-92% is associated with a lower risk of mortality, according to two small randomized trials [116].

Oxygen is delivered through the following devices during COPD exacerbations in hospitalized patients:

The nasal cannula provides flow rates of up to 6 L/min with FiO_2_ of 40%.

The venturi mask delivers FiO_2_ values of 24, 28, 31, 35, 40, and 60%. A higher limit of FiO_2_ may be preferred for patients with hypercapnic respiratory failure.

A simple face mask can provide a FiO_2 _of up to 55% with flow rates of 6 to 10 L/min.

Non-rebreathing masks can provide FiO_2_ of up to 90%, but they are generally not needed in COPD exacerbations.

The high-flow nasal cannula can provide oxygen with adjustable FiO_2_ at higher flow rates (up to 60 L/min). The indication of high-flow nasal cannula oxygen remains unclear in COPD exacerbation, and the comparison of high-flow nasal cannula with noninvasive ventilation in COPD exacerbation needs more research [11, 117, 118].

Patients should be assessed for the need for home oxygen within two days prior to discharge. This can be accomplished by measuring oxygen saturation at rest or during a six-minute walk. Patients with COPD often need long-term oxygen therapy (LTOT), defined as oxygen supplementation for at least 15 hours per day. Long-term oxygen therapy is prescribed if a patient has chronic severe hypoxemia, defined as an oxygen saturation <88%, a partial pressure of oxygen of <55 mmHg, an oxygen saturation of 89%, or a partial pressure of 56%-59% associated with cor pulmonale, right ventricular failure, or a hematocrit >55% [119]. Long-term oxygen therapy should be prescribed only when a patient has chronic, persistent hypoxia, is clinically stable, and is on goal-directed therapy for COPD. According to results from the nocturnal oxygen therapy trial (NOTT) and the medical research council (MRC) trial, LTOT increases survival in patients with severe chronic hypoxemia brought on by COPD [120, 121]. In COPD patients with chronic moderate hypoxemia, nocturnal oxygen therapy (NOT), additional oxygen for moderate hypoxemia, and LOTT revealed no effect [122, 123]. Table 10 summarizes LTOT trials in COPD.

**Table 10:** Randomized trials of long-term oxygen therapy in COPD Reproduced under the terms of the Creative Commons Attribution license. PaO_2_: partial pressure of oxygen; ECG: electrocardiography; aVF: augmented vector foot; FEV1: forced expiratory volume in 1 second; h/d: hours per day; LTOT: long-term oxygen therapy; PaCO_2_: partial pressure of carbon dioxide; SpO_2_: oxygen saturation; MWT: maintenance of wakefulness test [126]

Study	Inclusion Criteria	Intervention/Comparator	Primary Outcome	Main Results
Severe hypoxemia				
NOTT [120]	203 patients with COPD and PaO_2_ <55 mmHg, or PaO_2_ <59 mmHg with one of the following: 1) edema, 2) hematocrit >55%, or 3) P pulmonale on ECG: 3 mm in leads II, III, aVF.	Continuous oxygen (n = 101) vs. nocturnal oxygen (n = 102); frequent home and outpatient visits for both groups	Survival; adherence was monitored	At the two-year follow-up, the overall mortality of those receiving continuous oxygen was 22.4%, whereas it was 40.8% among those receiving nocturnal oxygen (P = 0.01)
MRC [121]	87 patients, 70 yr, FEV1 ,1.2 L, PaO_2_ between 40 and 60 mmHg.	Oxygen for >15 h/d (n = 42) vs. usual care (n = 45); some home visits and frequent outpatient visits in both groups.	Survival; adherence was not monitored.	At the five-year follow-up, 19 (45%) of 42 patients receiving oxygen therapy had died, compared with 30 (66%) of the 45 control patients.
Moderate hypoxemia				
G ´orecka et al. [123]	135 patients aged 40–80 yr, PaO_2_ 56–65 mm Hg	Oxygen for >17 h/d (n = 68) vs. usual care (n = 67)	Survival; adherence was monitored	Cumulative survival rates were 88%, 77%, and 66% at the one-, two-, and three-year follow-up, respectively. No significant difference in survival rates was seen between those receiving LTOT and the control group.
Haidl et al. [124]	28 patients with COPD and moderate hypoxemia (PaO_2_ at rest .55 mmHg) and reversible hypercapnia during the course of an exacerbation (PCO_2_ <45 mmHg at hospital discharge)	Oxygen for >15 h/d (n = 14) vs. usual care (n = 14)	Endurance time (cycle ergometry); survival; adherence was monitored	At the one-year follow-up, endurance time was improved among those receiving LTOT (7.1 vs. 4.9 min; P = 0.04); at the three-year follow-up, four patients receiving LTOT had died, compared with three control subjects.
LOTT [125]	738 patients with COPD and moderate resting desaturation (SpO_2_: 89–93%) or moderate exercise-induced desaturation (during 6MWT, SpO2 >80% for >5 min and 90% for >10s)	Continuous oxygen (or oxygen only during sleep and exercise in those with exercise-induced desaturation only) (n = 368) vs. usual care (n = 370)	Composite outcome of time to death or first hospitalization; compliance was monitored	Supplemental oxygen did not result in a longer time until death or first hospitalization than no long-term supplemental oxygen

Patients prescribed LTOT should be reassessed at three months to evaluate the ongoing need for LTOT and followed at six- to 12-month intervals [127]. Ambulatory oxygen therapy is defined as oxygen supplementation during exercise and daily activities in patients who become hypoxic during exertion. The American Thoracic Society (ATS) suggests using ambulatory oxygen for patients with severe exertional hypoxemia [128]. While the British Thoracic Society (BTS) does not recommend ambulatory oxygen therapy for isolated severe exertional hypoxemia, it should be prescribed to individuals who qualify for LTOT if they are mobile outdoors [129].

Extracorporeal Membrane Oxygenation 

**Figure 5:**
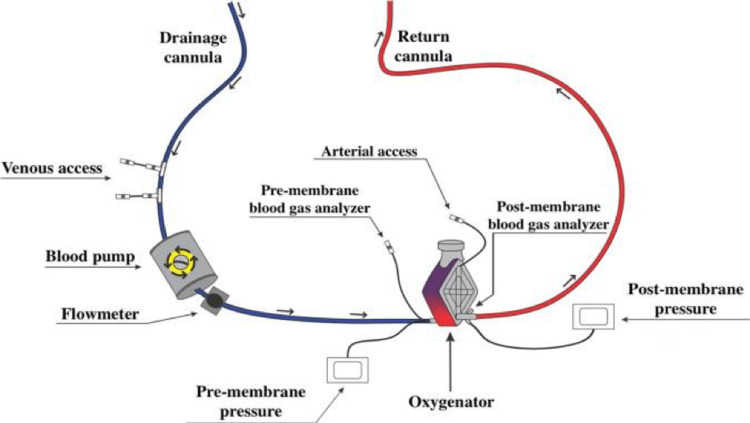
A typical extracorporeal membrane oxygenation circuit (ECMO) Reproduced under the terms of the Commons Creative Attribution license. [130]

Extracorporeal life support can be used to provide temporary support in respiratory failure [130-133] and/or cardiac failure [134-138], especially in cases where regular treatment has failed [130-138]. One of the most commonly used extracorporeal support devices is extracorporeal membrane oxygenation (ECMO) [139]. The venovenous-ECMO design is the most commonly preferred choice in respiratory failure with intact cardiac function [130-133]. The venoarterial-ECMO (VA-ECMO) design is utilized in cases of cardiac failure where lung function is either intact or not [134-138]. A typical extracorporeal membrane oxygenation circuit is depicted in Figure 5 below.

Through the use of a drainage cannula, the patient's venous blood is drawn out and pumped (blood pump) to the oxygenator. After passing through the oxygenator, which contains the oxygenation membrane, the blood is returned to the patient either through an artery (venoarterial-ECMO) or a vein (venovenous-ECMO). Along with pressure sensors (pre-membrane and post-membrane) and flow sensors, the ECMO circuit has access routes (venous and arterial access points) for the infusion of medications and fluids as well as a collection of laboratory tests.

Lung Volume Reduction Surgery (LVRS) 

**Figure 6:**
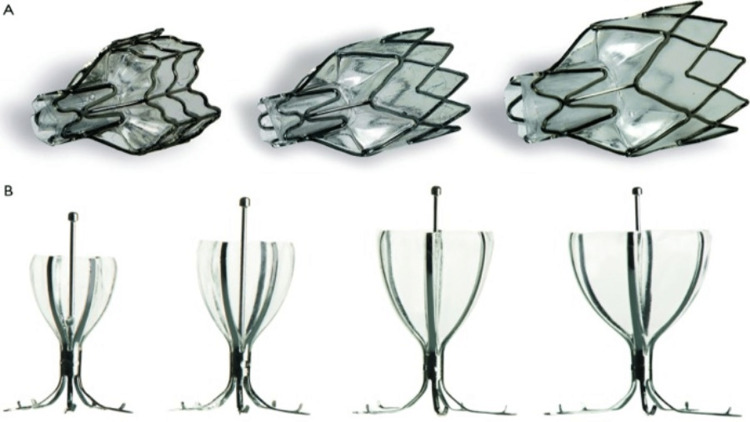
Endobronchial valves (Zephyr valve system, Pulmonx) (A) and intrabronchial valves (Spiration valve system, Spiration/Olympus) (B) Reproduced under the terms of the Creative Commons Attribution license. [148]

This is another procedure that can be used for severe emphysema or COPD [140]. In 2003, the National Emphysema Treatment Trial (NETT) was published, which was a large, randomized trial for LVRS involving multiple centers with a collaborative approach. This trial shed light on the impact of LVRS in providing benefits in survival ratios and improving the quality of life [141]. This trial's results currently help select patients for the LVRS procedure. Although this trial proved to be a monumental achievement for severe emphysema patients, further study was required and is still being conducted to assess the long-term outcomes of LVRS, cost efficiency, exploring LVRS as a potential path for definite lung transplantation, and unilateral vs. bilateral surgery [142-146]. There are many bronchoscopic lung reduction devices, but some common ones are foam sealants, metallic coils, airway bypass stents, endobronchial valves, and vapor thermal ablation. Coils are particularly useful for patients with severe emphysema and hyperinflation [147]. Figures 6A-6B below show endobronchial valves (Zephyr valve system, Pulmonx) and intrabronchial valves (Spiration valve system, Spiration/Olympus), respectively.

Endobronchial valves keep the air from re-entering the emphysematous regions, leading to their atelectasis. This makes it a valuable tool for bronchoscopic lung volume reduction [149]. The Endobronchial Valve for Emphysema Palliation (VENT) trial results, a significant study, showed only modest improvements in FEV1, dyspnea severity, distance covered in six minutes, and overall quality of life. But compared to regular care, this treatment method was associated with an increased risk of complications such as pneumonia and pneumothorax [150]. The details of the major studies that assessed the effectiveness of venous extracorporeal membrane oxygenation in treating patients with acute respiratory distress syndrome are presented in Table 11 below.

**Table 11:** Effectiveness of venous extracorporeal membrane oxygenation in treating patients with acute respiratory distress syndrome. MV: mechanical ventilation; PaO_2_: partial pressure of oxygen; FiO_2_: fraction of inspired oxygen; PaCO_2_: partial pressure of carbon dioxide; BMI: body mass index; VA-ECMO: venoarterial-extracorporeal membrane oxygenation; HIT: heparin-induced thrombocytopenia; RR: relative risk; CI: confidence interval; IS: ischemic stroke; PIP: peak inspiratory pressure; PEEP: positive end-expiratory pressure; PWP: pulmonary wedge pressure; ARDS: acute respiratory distress syndrome *Defined by the presence of a positive end-expiratory pressure ≥ 10 cmH2O and a tidal volume of 6 mL/kg of predicted weight

Author	No.'s	Design	Inclusion Criteria	Exclusion Criteria	Primary Outcome	Main Findings	Considerations
Combes et al. [130]	249	International multicenter, randomized, controlled clinical trial	1. Patient intubated on MV < 7 days 2. PaO_2_/FiO_2_ < 50mmHg for > 3 hours OR PaO_2_/FiO_2_< 80mmHg for > 6 hours OR Arterial pH < 7.25 with PaCO_2_ ≥ 60mmHg > 6 hours 3. Optimized MV^*^ 4. Age >18 years	1. Pregnant women 2. BMI > 45 3. Chronic respiratory failure 4. Indication for VA-ECMO 5. History of HIT 6. Advanced cancer 7. Dying patients 8. Coma after cardiac arrest 9. Nonreversible neurologic injury 10. Palliative patients	60-day mortality of 35% (44/124 patients) in the ECMO group and 46% (57/125 patients) in the control group (RR: 0.76; 95%CI 0.55 -1.04; p = 0.09)	Serious thrombocytopenia and bleeding that required transfusions were more common in the ECMO group. The incidence of IS, the requirement for renal replacement therapy, and the reduction of tidal volume, plateau pressure, and drive pressure were all lower in the ECMO group	Early interruption of the study due to futility Slow recruitment rate Due to refractory hypoxemia, the crossover rate was high (28%) as we switched from the control group to the ECMO group. At 60 days, the control group experienced a greater rate of treatment failure
Peek et al. [131]	180	Multicenter, randomized, controlled clinical trial	1. Age from 18 - 65 years 2. Severe but potentially reversible respiratory failure 3. Murray score ≥ 3.0 4. Uncompensated hypercapnia 5. Optimized MV.	1. PIP > 30cmH_2_O 2. FiO_2_ > 80% 3. Time of MV ≥ 7 days 4. Intracranial bleeding 5. Contraindication to heparinization 6. Limitation of support	6-month mortality after randomization or before hospital discharge of 37% (33/90) in the ECMO group and 53% (46/87) in the control group (RR: 0.69; 95%CI 0.05-0.97, p = 0.03)	The transfer of patients with severe but potentially reversible respiratory failure to a reference center in ECMO proved to be cost-effective and reduced mortality	Control group does not have standardization of MV parameters 22 patients did not use the device of the 90 patients who were randomized to receive ECMO
Morris et al. [133]	40	Dual-center, randomized, controlled clinical trial	1. PaO_2_ < 50mmHg for 2 hours with FiO_2_ = 100%, PEEP > 5 and PaCO_2_ of 30 - 45 or PaO_2_ < 50mmHg for 12 hours with FiO_2_ = 60%, PEEP ≥ 5cmH_2_O and PaCO_2_of 30 - 45 2. Optimized MV	1. Contraindication to anticoagulants 2. POAP > 25mmHg 3. Time of MV> 21 days 4. Severe, irreversible, and without treatment prospective systemic disease	30-day survival of 33% (7/21) in the ECMO group and 42% (8/19) in the control group (p = 0.8)	Does not advocate the use of ECMO in ARDS patients.	Small sample size High mortality rate (62% of patients died) Technical limitations inherent to the clinical trial period Non-protective MV in both groups
Zapol et al. [132]	90	Multicenter, randomized, controlled clinical trial	1. PaO_2_ < 50 mm Hg, for more than 2 hours with FiO_2_100% and PEEP ≥ 5cmH_2_O OR PaO_2_ < 50 mm Hg, for more than 12 hours with FiO_2_ = 60% and PEEP ≥ 5cmH_2_O	1. Age from 12 to 65 years old 2. Pulmonary lesion time > 21 days 3. PWP > 25mmHg 4. Severe, irreversible, and incurable systemic disease	30-day survival of 9.5% (4/42) in the ECMO group and 8.3% (4/48) in the control group (no significant difference)	The survival rate of patients with severe ARDS was not improved by ECMO, but it was able to support the respiratory system.	Mortality in both groups is greater than 90%. Technical limitations were inherent to the clinical trial period. Nonprotective MV in both groups.

People with incurable, advanced lung disease who have a short life expectancy are given the option of a lung transplant. A double lung transplant is most commonly done for severe COPD patients [150]. The International Society for Heart and Lung Transplantation (ISHLT) dictates the criteria for referral of COPD patients for lung transplantation; it includes an FEV1 ≤ 30% despite being on maximum pharmacological and non-pharmacological therapy, including bronchodilators, home oxygen, smoking cessation, and pulmonary rehabilitation. Additionally, a BMI, Obstruction, Dyspnea, and Exercise Capacity (BODE) index >5 [151] is important in timing the listing of patients for a lung transplant. It is also used as an indicator of death in COPD patients. Unfortunately, COPD patients receiving transplants do not see the same significant improvement in their survivability as those with other conditions such as idiopathic pulmonary fibrosis, cystic fibrosis, and primary pulmonary hypertension [152]. However, COPD patients have improved respiratory function, endurance, and quality of life [152].

**Figure 7:**
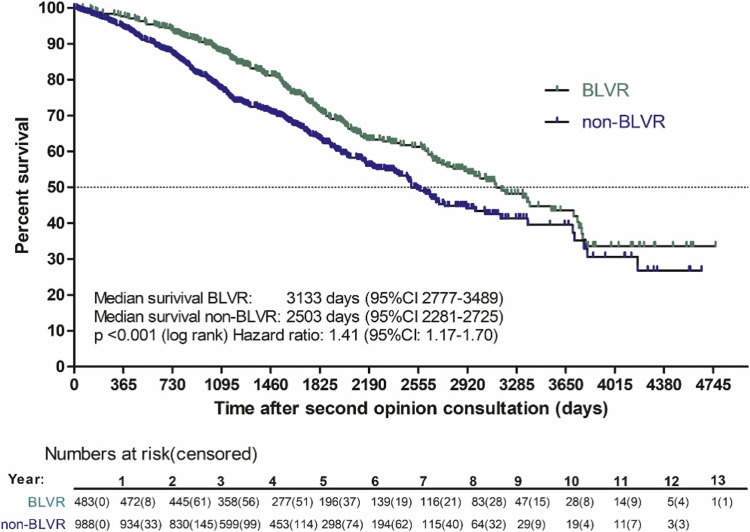
Kaplan-Meier plot of survival of patients who underwent BLVR treatment and who did not. Reproduced under the terms of the Commons Creative Attribution License. BLVR: bronchoscopic lung volume reduction; non-BLVR; non-bronchoscopic lung volume reduction; CI: confidence interval *BLVR: patients who underwent a bronchoscopic lung volume reduction treatment. Non-BLVR: patients who did not undergo a bronchoscopic lung volume reduction treatment. The confidence interval was 95%. [133]

Figure 7 below illustrates a Kaplan-Meier plot of the survival of patients who underwent a bronchoscopic lung volume reduction (BLVR) treatment instead of those that did not.

**Figure 8:**
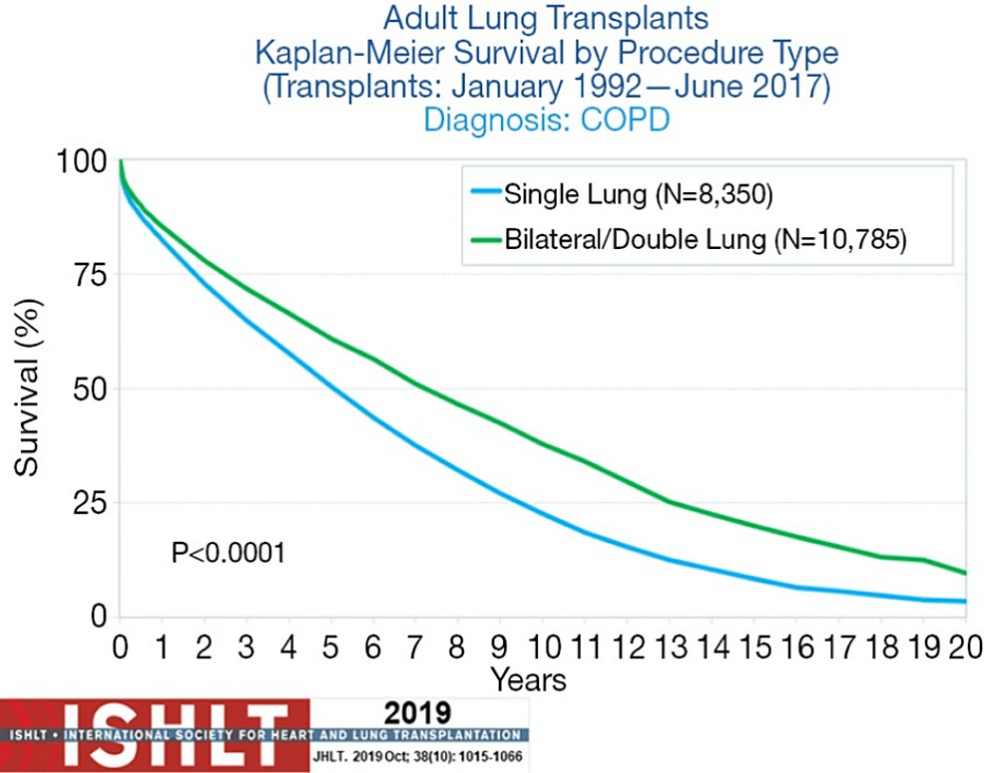
Thirty-sixth Adult Lung and Heart-Lung Transplantation Report (2019) of the International Thoracic Organ Transplant Registry (ITOTR) of the International Society for Heart and Lung Transplantation (ISHLT) Reproduced under the terms of the Creative Commons Attribution license. [132]

In a study by Hartman et al., a total of 1471 patients were included. Patients were predominantly female (63%) with a mean age of 61 years, a 40-pack-year smoking history, low FEV1 (30%), hyperinflation with RV (221% predicted), an emphysema destruction score of 36.8% (−950 Hounsfield Units (HU)), and a St. George’s Respiratory Questionnaire (SGRQ) total score of 59 units [153]. In the recent International Registry (ISHLT) illustrated in Figure 8, median survival for COPD recipients who received bilateral or single lung transplants was 7.8 years versus 4.8 years for single lung transplant recipients.

Survival rates were calculated using the Kaplan-Meier method. The log-rank test statistic was used to compare survival rates.

The University of Michigan healthcare staff has thoroughly penned a checklist on the key elements of hospital discharge for patients with AECOPD (Table 12).

**Table 12:** The key elements of hospital discharge for patients with AECOPD Reproduced under the terms of the Creative Commons Attributions license. CHF: congestive heart failure; CMS: Centers for Medicare and Medicaid Services; O_2_: oxygen; HTN: hypertension; LE: lower extremity; PFT: pulmonary function testing; FEV1: forced expiratory volume in one second; FVC: forced vital capacity; AECOPD: acute exacerbation of chronic obstructive pulmonary disease [154]

Key element	Notes
Ensure the patient’s discharge readiness.	The patient’s dyspnea should be improved to the point that the patient can eat, sleep, walk, and correctly use inhaler medications (assuming that the patient was able to do these things at baseline). The patient should be clinically stable for 12-24 hours, with short-acting bronchodilators required no more frequently than every four hours.
Ensure an appropriate COPD medication regimen at discharge.	Complete antibiotic and corticosteroid courses, if initiated in the hospital. An inhaler regimen should include, at minimum, a long-acting bronchodilator and a rescue inhaler. Consider inhaled corticosteroids and prophylactic antibiotics on a case-by-case basis.
Ensure patient education about critical elements of care, including correct inhaler technique.	All patients should be educated on the importance of adherence to the inhaler regimen, and correct inhaler technique, and counseled on smoking cessation if appropriate.
Assess the patient’s need for home O_2_.	Home O_2_ should be provided if the patient meets the following criteria: Room air O_2_ saturation < 89% (or pO_2 _<56), or room air O_2_ saturation of 89% (or pO_2_ of 56–59) + one of the following three features: LE edema is suggestive of CHF, pulmonary HTN/cor pulmonale, or erythrocytosis (hematocrit > 56%).
Refer eligible patients for pulmonary rehabilitation.	CMS requires PFTs to demonstrate at least moderate disease (FEV1/FVC < 0.70 and FEV1 <80% predicted) to qualify for pulmonary rehabilitation.
Ensure that the patient has appropriate, timely follow-up.	At the time of discharge, consider the use of an assessment tool to estimate readmission risk. Follow-up in a clinical setting familiar with transitional care can be beneficial. Schedule full PFTs (including bronchodilators) four to six weeks after AECOPD, if not previously performed.

## Conclusions

Managing COPD exacerbations in hospitalized patients is a complex and multifaceted process. The management strategies outlined in this literature, including inhaled bronchodilators, systemic steroids, antibiotics, oxygen therapy, and ventilation modes, have improved clinical outcomes and reduced mortality rates. However, the choice of ventilation modality should be tailored to the individual patient's needs and clinical status. Additionally, it has been shown that vaccination, pulmonary rehabilitation, and smoking cessation all enhance patient outcomes and decrease the frequency of exacerbations. The last resort in treating COPD exacerbations is lung transplantation, which is not a permanent cure as the five-year survival rate is only about 50%. Keeping this in mind, it can be concluded that there is still room for improvement in this disease's treatment and overall prognosis, particularly in exacerbations.
